# Coping in the Face of Verbal Aggression: The Role of Self-Efficacy in Protecting Healthcare Professionals’ Well-Being and Job Satisfaction

**DOI:** 10.3390/bs15040478

**Published:** 2025-04-06

**Authors:** Elena Cavallari, Ilaria Setti, Matteo Curcuruto, Valentina Sommovigo

**Affiliations:** 1 Unit of Applied Psychology, Department of Brain and Behavioural Sciences, University of Pavia, 27100 Pavia, Italy; elena.cavallari01@universitadipavia.it (E.C.); ilaria.setti@unipv.it (I.S.); 2 Department of Human Sciences, European University of Rome, 00163 Rome, Italy; matteo.curcuruto@unier.it; 3 Department of Psychology, Faculty of Medicine and Psychology, Sapienza, University of Rome, 00185 Rome, Italy

**Keywords:** user verbal aggression, healthcare workers, psychological withdrawal responses, self-efficacy, job satisfaction, workload

## Abstract

Verbal aggression toward healthcare professionals, primarily from patients and visitors, is widespread and negatively affects employee well-being and patient care quality. This study, comprising two samples, investigates the relationship between user-initiated verbal aggression and job satisfaction, with a focus on psychological processes (i.e., cynicism and mental distance) and personal boundary conditions (i.e., self-efficacy). Study 1 (pandemic period) explored cynicism and work-related self-efficacy, while Study 2 (post-pandemic) replicated and expanded these findings, incorporating mental distance, self-efficacy in managing negative emotions, and workload. Participants included 201 (Study 1) and 1442 (Study 2) healthcare professionals from one and eight Italian healthcare facilities, respectively, who completed online questionnaires. In both cross-sectional studies, verbal aggression was positively associated with psychological withdrawal responses, which, in turn, was negatively related to job satisfaction. However, high self-efficacy in managing negative emotions (rather than work-related self-efficacy) buffered these effects. In Study 2, the negative impact of verbal aggression on job satisfaction, mediated by mental distance, was most pronounced among those with low self-efficacy in managing negative emotions and a high workload. Conversely, individuals with high self-efficacy maintained their job satisfaction and did not exhibit psychological withdrawal, even under high workload conditions.

## 1. Introduction

Imagine yourself in the relentless flow of a bustling hospital emergency department. Every shift brings a steady stream of patients, many visibly frustrated by long waits and limited resources. Family members demand answers, and after hours of waiting, patients direct their anger at you. As you approach, they greet you not with relief but with sharp words and accusations, holding you personally responsible for their delay. In an attempt to shield yourself, you begin emotionally withdrawing. Could this affect how much you enjoy your job? What if you do not consider yourself particularly good at your job or adept at managing your emotions—could this make it harder for you to handle verbal aggression from users (i.e., patients and visitors)? And with a growing number of patients, could an overwhelming workload make it even more difficult to cope with these stressors, deepening your dissatisfaction with your job?

**Figure 1:**
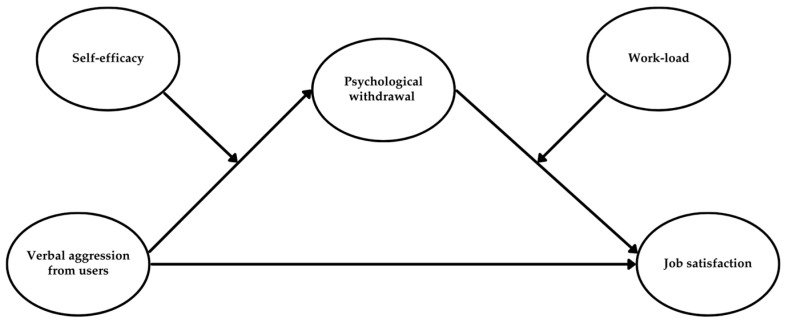
Overall conceptual model representation.

This study aims to address these questions by testing a conceptual model that examines whether usual verbal aggression is negatively associated with job satisfaction via psychological withdrawal responses. Additionally, it investigates whether this link is moderated by self-efficacy and workload (see Figure 1).

In recent decades, healthcare professionals have faced a concerning increase in aggressive behavior from users (i.e., patients, family members, and/or visitors; Mento et al., 2020). The World Medical Association (2021) has classified this issue as “an international emergency that undermines the very foundations of health systems and critically impacts patient health.” Among various types of workplace aggression, verbal aggression is the most prevalent, with approximately 97% of healthcare workers reporting exposure, primarily from users (Berger et al., 2024). Verbal aggression includes hostile and intentionally harmful expressions, including threats, insults, and shouting (McLaughlin et al., 2010). The impact of verbal aggression extends beyond immediate psychological distress. Repeated exposure to hostile verbal interactions is associated with increased levels of depression, anxiety, and emotional exhaustion—key precursors to burnout (Myers & Kowal, 2024). This psychological burden can impair attentiveness, hinder decision making, and elevate the risk of medical errors, ultimately compromising patient care (Willer et al., 2023; Pacheco et al., 2021). At the organizational level, sustained verbal aggression contributes to higher absenteeism, staff turnover, and diminished team cohesion, further exacerbating workforce shortages and operational inefficiencies (López-Ros et al., 2023).

Even prior to COVID-19, verbal aggression posed significant challenges, but the pandemic exacerbated these issues (Byon et al., 2021). Exhausting shifts and increased psychological pressures became routine as healthcare professionals faced fears of infection, uncertainty, and restrictive protocols, all of which heightened user frustration (Sommovigo et al., 2022b). The crisis acted as a catalyst for verbal aggression: safety protocols like social distancing and restricted access reduced instances of physical aggression but contributed to an increase in verbal incidents (Rossi et al., 2023; Brigo et al., 2022). Despite the end of the acute health emergency, verbal aggression remains widespread as the healthcare system continues to face backlogs and staff shortages, perpetuating these incidents (Rossi et al., 2023; Brigo et al., 2022).

The Conservation of Resources theory (Hobfoll, 1989) provides a valuable framework for understanding how verbal aggression from users depletes healthcare professionals’ resources. This theory posits, among other things, that individuals are fundamentally motivated to acquire, preserve, and safeguard resources—broadly defined as objects, personal characteristics, conditions, and energies valued either intrinsically or instrumentally (Hobfoll et al., 2018, 2023). According to this framework, psychological stress arises when there is a threat to these resources, an actual loss, or an insufficient return on resource investment. A key tenet of the Conservation of Resources theory is the primacy of resource loss, which posits that losses are experienced more intensely than equivalent gains, leading individuals to exhibit heightened sensitivity to potential or actual resource depletion (Hobfoll et al., 2023; Demerouti, 2025). Within this perspective, verbal aggression represents a significant threat to healthcare workers’ resource systems (Sommovigo et al., 2022b). Such hostile encounters often occur after considerable emotional and cognitive investment in maintaining professional behavior during challenging social interactions (e.g., offering empathic care or de-escalating tense situations; Sommovigo et al., 2024). When these efforts are met with incivility or hostility, individuals may perceive a loss of anticipated resource gains (e.g., appreciation and cooperation) and experience direct threats to critical personal (e.g., self-esteem and emotional stability) and social (e.g., perceived support and relational trust) resources (Sommovigo et al., 2022b). Consequently, verbal aggression undermines immediate well-being and can initiate a downward spiral of resource depletion, impairing future coping capacity and job-related satisfaction (Sommovigo et al., 2022b).

Drawing on the Conservation of Resources theory (Hobfoll, 1989), verbal aggression from users is a significant work stressor that depletes essential resources, such as energies (e.g., time), personal resources (e.g., self-esteem), and valued social conditions (e.g., feeling safe at work; Sommovigo et al., 2022b). This drives healthcare professionals to adopt defensive strategies to conserve what remains (Hobfoll et al., 2018; Demerouti, 2025). These resource-conservation strategies often result in psychological withdrawal responses (Vidal-Alves et al., 2022). Core facets of psychological withdrawal in high-pressure environments include cynicism (i.e., emotionally detached responses toward service recipients; Montgomery & Maslach, 2019) and mental distance (i.e., detachment from work, often accompanied by negative attitudes toward job responsibilities; Schaufeli & De Witte, 2023). Such detachment responses can worsen burnout among healthcare professionals, compromise adherence to ethical and professional standards, and ultimately diminish patient satisfaction (Cao et al., 2023; Hodkinson et al., 2022). Furthermore, these responses can impair clinical decision making, reduce teamwork effectiveness, and increase the likelihood of medical errors, thereby posing a direct risk to patient well-being (Cao et al., 2023; Hodkinson et al., 2022). This cycle perpetuates burnout, reduces professionalism, and lowers patient satisfaction (Cao et al., 2023; Hodkinson et al., 2022; Vidal-Alves et al., 2022). Moreover, job satisfaction—a holistic evaluation of one’s role that encompasses both cognitive and emotional dimensions—may suffer as healthcare professionals find their sense of purpose and fulfillment eroded by these coping mechanisms (Briganti et al., 2023). According to the World Health Organization (2024), the well-being and job satisfaction of healthcare workers are critical for delivering high-quality patient care. Improving job satisfaction and optimizing working conditions are essential for sustaining the healthcare workforce, particularly given the increasing demands and ongoing staff shortages (World Health Organization & International Labour Organization, 2022). Ensuring decent working conditions in the healthcare sector is vital for building effective and resilient health systems (International Labour Organization, 2022). It is a critical prerequisite for addressing workforce shortages and advancing equitable access to high-quality healthcare (International Labour Organization, 2022). Empirical evidence consistently indicates that enhancing job satisfaction not only improves the well-being of healthcare professionals but also leads to better patient outcomes, higher staff retention, and improved overall organizational performance and climate (Karaferis et al., 2022). Examining the psychological mechanisms that link aggression to job dissatisfaction can provide insights into strategies aimed at supporting healthcare workers and fostering job satisfaction, which is vital for improving patient outcomes (Briganti et al., 2023).

According to the Conservation of Resources theory, individuals rely on various forms of resources to manage stressful events, including personal (e.g., self-efficacy and emotion regulation skills) and social (e.g., social support and positive relationships with users) resources. Healthcare professionals who possess a robust reservoir of personal and social resources tend to be more resilient to workplace stressors (Goussinsky, 2020, 2024; Goussinsky & Livne, 2019; Gilardi et al., 2020). Conversely, employees with limited access to resources—such as those lacking social support or experiencing low self-confidence in handling emotional or task-related demands—are more vulnerable to the detrimental effects of stressors (Goussinsky, 2020, 2024; Goussinsky & Livne, 2019). Complementing this, the Social Cognitive theory (Bandura, 2023) suggests that self-efficacy beliefs interact with the work environment to influence how, why, and when employees react to environmental stimuli. Integrating the Conservation of Resources theory with the Social Cognitive theory, self-efficacy serves as a key personal resource that enables employees to self-regulate in response to relevant challenges (Bandura, 2023; Hobfoll et al., 2015). Compared to general self-efficacy, domain-specific self-efficacy provides a more accurate prediction of cognitive responses and behaviors in specific settings (Bandura, 2023).

Work-related self-efficacy, in particular, refers to an individual’s confidence in their ability to successfully perform job-related tasks and fulfill professional responsibilities (Luthans et al., 2007). Another key factor in user-facing roles is emotional regulation, which is crucial for managing interpersonal interactions and maintaining professionalism even in challenging situations (M. Caprara et al., 2023). These roles often require adherence to “display rules”—e.g., maintaining a positive demeanor with users regardless of circumstances (M. Caprara et al., 2023). Managing these emotional demands requires substantial resource investment (Hobfoll et al., 2018, 2023). Accordingly, this study examines regulatory self-efficacy in managing negative emotions (RESE-NE, G. V. Caprara et al., 2008). RESE-NE refers to individuals’ cognitive appraisals of their capacity to recover from and manage adverse emotional states triggered by work-related stressors (Alessandri et al., 2015a, 2022). RESE-NE plays a pivotal role in shaping how individuals interpret and respond to emotionally challenging situations, facilitating their ability to swiftly regain emotional equilibrium and confidently manage workplace stressors, such as verbal aggression from users (Alessandri et al., 2015a, 2022; Bandura, 1997, 2023; G. V. Caprara et al., 2008). Individuals with higher levels of RESE-NE tend to see interpersonal difficulties as manageable challenges, as their ability to regulate negative emotions relies on their belief in their own capacity to do so (G. V. Caprara et al., 2008). As a result, employees with strong RESE-NE are more likely to respond constructively, preserving the emotional energies necessary for meaningful professional interactions and job satisfaction (Kwak et al., 2020; Madrid et al., 2020).

According to the Conservation of Resources theory, personal resources become especially vital in resource-poor environments, where external resources (e.g., opportunities for recovery, social support, or institutional assistance) are scarce or unreliable (Hobfoll et al., 2015; Demerouti, 2025). In such situations, employees must rely more heavily on their internal coping resources to navigate ongoing demands. However, without adequate replenishment, these internal resources can become depleted, increasing individuals’ susceptibility to stress (Headrick et al., 2022). In healthcare settings, workload—the volume and intensity of tasks required within a specified timeframe (Broetje et al., 2020)—emerges as a critical and often overwhelming stressor (Eurofound, 2017). This workload includes various demands, such as patient volume, administrative duties, time constraints, and the complexity of medical cases (Eurofound, 2017). The COVID-19 pandemic has further compounded these challenges by increasing workload while limiting opportunities for recovery from physical exhaustion, emotional strain, and cognitive overload (Marzocchi et al., 2024). Although the immediate impacts of the pandemic have lessened, excessive workload remains a persistent source of stress for healthcare professionals, with increasing evidence suggesting that they experience lower job satisfaction under such conditions (Doleman et al., 2023).

By investigating psychological withdrawal responses as the psychological processes that connect user verbal aggression to job dissatisfaction and identifying the personal and contextual boundary conditions that jointly shape this relationship, this study offers a more nuanced understanding of how healthcare professionals respond to verbal aggression from users (see Figure 1). The research illuminates both individual (self-efficacy) and environmental (workload) factors that shape these responses, highlighting the intricate interplay of these factors. As such, the study provides valuable insights for healthcare organizations to develop targeted interventions that help healthcare workers maintain satisfaction even in the face of user verbal aggression.

### 1.1. How Is Verbal Aggression Related to Job Satisfaction in Healthcare?

According to the Conservation of Resources theory (Hobfoll, 1989), individuals strive to acquire, retain, and safeguard valuable resources, becoming increasingly sensitive to resource loss. For healthcare workers, verbal aggression from users threatens their energies (e.g., time), personal resources (e.g., self-esteem), and valued social conditions (e.g., feeling safe at work; Sommovigo et al., 2022b). Such aggression hinders healthcare professionals’ ability to restore essential resources despite their ongoing efforts (Hobfoll et al., 2018, 2023). Repeated exposure to verbal aggression from users depletes resources and increases feelings of helplessness, anxiety, and stress, often leading to job dissatisfaction (Cao et al., 2023; Lim et al., 2022). Additionally, verbal aggression disrupts the satisfaction of basic psychological needs (e.g., competence and relatedness), which amplifies powerlessness and further reduces job satisfaction (Goussinsky & Livne, 2019). Empirical studies indicate a negative association between user mistreatment and job satisfaction, both directly and indirectly (Briganti et al., 2023; Cao et al., 2023; Stafford et al., 2022), with verbal aggression having a particularly strong correlation with dissatisfaction compared to other forms of mistreatment (Han et al., 2021). Based on this, we propose the following hypothesis:

**Hypothesis** **1:** *User verbal aggression will be negatively related to job satisfaction.*

### 1.2. Psychological Withdrawal Responses Linking User Verbal Aggression to Job Satisfaction

Research consistently demonstrates a strong link between user non-physical aggression and healthcare providers’ psychological withdrawal responses (Goussinsky, 2020, 2024; Goussinsky & Livne, 2019). Cynicism is characterized by emotional detachment, a diminished sense of purpose, and reduced concern for patient care—often as a defensive response to interpersonal stressors, particularly in high-pressure environments like healthcare (Pina et al., 2022; Toscanelli et al., 2023). In contrast, mental distance involves a conscious effort to emotionally and cognitively withdraw from work, allowing individuals to “shut off” from tasks and social interactions as a coping mechanism for stress (Schaufeli & De Witte, 2023). While both constructs involve detachment, cynicism is more closely associated with emotional disengagement and loss of meaning in work. In contrast, mental distance refers to a broader cognitive and emotional withdrawal aimed at conserving personal resources in response to occupational demands (Schaufeli & De Witte, 2023). Both cynicism and mental distance serve as psychological withdrawal mechanisms that may provide temporary relief from emotional strain but can also reduce empathetic engagement with patients and decrease job satisfaction (Hodkinson et al., 2022).

Drawing on the Conservation of Resources theory (Hobfoll et al., 2018), verbal aggression from users threatens healthcare providers’ valuable resources (Sommovigo et al., 2022b). When providers repeatedly encounter aggression and have limited opportunities for resource recovery (e.g., through restorative practices), they may experience resource depletion (Hobfoll et al., 2018). This depletion can trigger self-protective responses, reducing their capacity for meaningful patient engagement and increasing the likelihood of adopting psychological withdrawal responses as coping strategies (Schaufeli & De Witte, 2023). Although these responses may be self-protective, they can also lower care quality and contribute to dissatisfaction among providers, eroding their sense of purpose and fulfillment (Toscanelli et al., 2023). Given these distinctions, Study 1 examined cynicism as a key dimension of burnout, establishing a foundation for understanding emotional disengagement from verbal aggression. Meanwhile, Study 2 explored mental distance, a construct aligned with contemporary perspectives on adaptive coping mechanisms in high-demand work environments. This twofold approach highlights different aspects of psychological withdrawal and their implications for healthcare professionals’ well-being and job satisfaction. Therefore, we propose the following hypotheses:

**Hypothesis** **2:** *User verbal aggression will be positively related to withdrawal responses (Hp2a: cynicism; Hp2b: mental distance).*

**Hypothesis** **3:** *The negative relationship between user verbal aggression and job satisfaction will be mediated by psychological withdrawal responses (Hp3a: cynicism; Hp3b: mental distance).*

### 1.3. Protective Power of Personal Resources: How Does Self-Efficacy Shield Against User Verbal Aggression?

According to the Conservation of Resources theory, certain personal characteristics, such as resilience and emotional competencies (Hussain et al., 2022; Hirschle & Gondim, 2020) serve as essential resources that enable individuals to withstand and manage stress (Hobfoll et al., 2018). These characteristics function as intrinsic coping mechanisms, and their varying strengths influence how effectively individuals respond to adversity (Hobfoll et al., 2015; Demerouti, 2025). Individuals with strong personal resources are better equipped to minimize losses and handle stressors, while those with fewer resources are more vulnerable to resource depletion (Hobfoll et al., 2023). Empirical studies underscore the significance of personal resources in sustaining job satisfaction, especially in high-stress fields like healthcare, where they shape employees’ responses to stressors such as challenging patient interactions (Haehnle et al., 2022). Workers with stronger resources often report higher job satisfaction (Marzocchi et al., 2024).

Self-efficacy is a key personal resource for managing workplace challenges (Bandura, 2023; Wu et al., 2024). Based on the Social Cognitive theory, self-efficacy reflects individuals’ perceptions of their control over their actions and environments, influencing their capacity to handle demands, self-regulate, and respond to stressors (Bandura, 2023). The Social Cognitive theory posits that self-efficacy is domain-specific and varies across different life contexts (Bandura, 2023). This research examines two types of self-efficacy: work-related self-efficacy, which concerns confidence in managing work-related tasks (Luthans et al., 2007), and RESE-NE, which reflects confidence in recovering from and managing negative emotions triggered by work-related adversities (Alessandri et al., 2015b, 2022). RESE-NE is vital for how individuals perceive and respond to distressing events, supporting quicker recovery from negative emotions and effective emotion regulation at work (Alessandri et al., 2015b, 2022). High RESE-NE enables individuals to view stressful situations as manageable, bolstered by confidence in their ability to learn from adversity (Bandura, 2023). Furthermore, the Social Cognitive theory suggests that self-efficacy shapes social perceptions, with high RESE-NE promoting positive interpretations of others’ behaviors and fostering constructive workplace interactions (Bandura, 2023).

We anticipate that healthcare professionals with high self-efficacy—whether related to job tasks or managing negative emotions—will maintain a constructive outlook despite frequent verbal aggression. They are likely to perceive these interactions as manageable, which can reduce the need for psychological withdrawal responses and enhance their commitment to patient care (Marzocchi et al., 2024). By upholding their sense of purpose, self-efficacious healthcare professionals will be better positioned to maintain job satisfaction, even in the face of verbal aggression (Haehnle et al., 2022; Parent-Lamarche & Fernet, 2020). Thus, we propose the following hypotheses:

**Hypothesis** **4:** *Self-efficacy (Hp4a: work-related self-efficacy and Hp4b: RESE-NE) will moderate the positive relationship between user verbal aggression and psychological withdrawal responses (i.e., cynicism or mental distance). Specifically, healthcare professionals with higher levels of self-efficacy are expected to be less likely to adopt these withdrawal responses when facing user verbal aggression.*

**Hypothesis** **5:** *The negative indirect effect of user verbal aggression on job satisfaction, mediated by psychological withdrawal responses (i.e., cynicism or mental distance), will be weakened for healthcare professionals with high self-efficacy (Hp5a: work-related self-efficacy and Hp5b: RESE-NE). Greater self-efficacy will help healthcare professionals maintain higher job satisfaction despite exposure to user verbal aggression.*

### 1.4. The Impact of Job Demands: The Moderating Role of Workload

According to the Conservation of Resources theory (Hobfoll, 1989), in low-resource environments—such as those characterized by excessive workloads—individuals must exert more effort to counteract resource depletion and restore lost resources. When psychological withdrawal is used as a resource-conservation strategy in response to user aggression under conditions of excessive workload, it can reduce professionals’ sense of purpose and intrinsic motivation (Broetje et al., 2020; Marzocchi et al., 2024; Toscanelli et al., 2023). This emotional distancing can further disconnect them from meaningful patient interactions and work fulfillment (Broetje et al., 2020; Marzocchi et al., 2024; Toscanelli et al., 2023). This compounded resource drain limits professionals’ capacity to engage in restorative activities that could replenish their emotional resources (e.g., supportive colleague interactions or breaks; Broetje et al., 2020; Marzocchi et al., 2024). Moreover, high workloads typically reduce control over one’s work environment, leading to frustration, which makes psychological withdrawal a more ingrained response (Doleman et al., 2023). This can result in job dissatisfaction, as individuals may feel increasingly detached and unable to engage fully in aspects of their work that contribute to their satisfaction (Marzocchi et al., 2024). Conversely, a manageable workload allows for recovery following aggressive encounters, enabling resource-restorative activities, such as positive social interactions, that help replenish emotional and cognitive reserves (Marzocchi et al., 2024). This process enables healthcare professionals to manage verbal aggression more effectively, restoring their sense of control over their work environment (Hobfoll et al., 2018, 2023). Additionally, a lighter workload facilitates more personalized patient care, enhancing the sense of purpose and protecting against job dissatisfaction (Marzocchi et al., 2024; Wu et al., 2024). Accordingly, we propose the following hypotheses:

**Hypothesis** **6:** *Workload will moderate the relationship between mental distance and job satisfaction, such that healthcare professionals experiencing high (versus low) workload will be more likely to report lower job satisfaction.*

**Hypothesis** **7:** *The negative indirect effect of user verbal aggression on job satisfaction, mediated by mental distance, will be exacerbated when the workload is high.*

### 1.5. Overall Moderated Mediation Model

Our integrated moderated mediation framework (see Figure 1) suggests that self-efficacy and workload interactively moderate the pathway from user verbal aggression to job satisfaction, with mental distance serving as a mediator. Specifically, we expect that RESE-NE will buffer the positive association between aggression and mental distance, while workload will exacerbate the negative association between mental distance and job satisfaction. Thus, the entire indirect pathway from aggression to job satisfaction is anticipated to be conditional on the combined moderating effects of RESE-NE and workload. Building on our prior arguments, we propose that healthcare professionals with lower RESE-NE will be more vulnerable to resource depletion, especially under high workload conditions (Wu et al., 2024). This reasoning suggests that the negative indirect effects of aggression on job satisfaction, mediated by mental distance, will be more pronounced when RESE-NE is low and workload is high. Thus, we propose the following hypothesis:

**Hypothesis** **8:** *RESE-NE and workload will jointly moderate the link between user verbal aggression and job satisfaction through mental distance, such that this negative indirect effect will be stronger when healthcare professionals have lower self-efficacy and higher workloads.*

### 1.6. Overview of the Current Research

Previous researchers have suggested that studies involving multiple investigations can significantly enhance the literature through replication and extension (Montani et al., 2020; Morgan et al., 2021). Similarly, scholars have advocated for the examination of theoretical models, or their components, through improved or diverse independent empirical approaches (Cortina et al., 2017). Following these recommendations and consistent with prior research (e.g., Bernuzzi et al., 2024; Montani et al., 2020), we conducted two studies investigating the relationship between user verbal aggression, psychological withdrawal responses, and job satisfaction. Study 1 used a cross-sectional design with a sample of 201 Italian healthcare workers during the pandemic, aiming to investigate whether cynicism mediates the anticipated negative relationship between patient verbal aggression and job satisfaction, and whether this relationship is conditional on work-related self-efficacy. Given the pandemic’s unique circumstances that limited hospital access, Study 1 focused exclusively on verbal aggression from patients. Study 2 aimed to replicate the findings of Study 1 in a post-pandemic context using a larger sample of 1442 healthcare professionals from eight hospitals, while also extending the previous results to include verbal aggression from both patients and visitors (i.e., users). This study also investigated the joint moderating role of RESE-NE and workload.

Study 1 explored cynicism as a core dimension of burnout, offering a framework for understanding emotional disengagement in response to verbal aggression. In contrast, Study 2 examined mental distance, a construct that aligns with contemporary perspectives on adaptive coping in high-stress work environments. This dual-framework approach clarifies distinct forms of psychological withdrawal, offering a more comprehensive understanding of their impact on healthcare professionals’ job satisfaction. Additionally, the shift from work-related self-efficacy to RESE-NE reflects a broader transformation in healthcare challenges, transitioning from acute crisis management to long-term adaptation. During the pandemic, healthcare professionals faced a multitude of occupational stressors, including extended work shifts, stringent safety protocols, shortages of personal protective equipment, unclear treatment guidelines, and the pervasive fear of infection and transmission (Maffoni et al., 2022). At the height of the crisis, they relied on their confidence in technical competencies and problem-solving abilities to address these evolving medical and logistical demands (Maffoni et al., 2022). However, as immediate operational disruptions subsided, the residual psychological burden became increasingly salient. In this new context, individuals with higher RESE-NE are better equipped to maintain professional functioning and psychological well-being, making it a more relevant and adaptive form of self-efficacy in the post-pandemic period. Consequently, our decision to assess RESE-NE instead of work-related self-efficacy in Study 2 reflects this shift in workplace stressors within healthcare settings. Evaluating RESE-NE offers a clearer measure of essential psychological resources for healthcare professionals facing ongoing challenges. Moreover, the inclusion of workload in Study 2 reflects the recognition that as the crisis evolved, the sheer volume and intensity of work—alongside the associated emotional demands—became the key factors influencing job satisfaction (Rossi et al., 2023). By exploring both dimensions of burnout and evolving constructs of self-efficacy, these studies work together to provide a comprehensive understanding of workplace stressors in healthcare. Study 1 lays the groundwork by examining emotional disengagement through cynicism. In contrast, Study 2 builds on this foundation by introducing the concept of mental distance and investigating the combined moderating effects of RESE-NE and workload. This approach offers a more detailed view of coping mechanisms and the psychological resources that support healthcare professionals.

## 2. Materials and Methods: Study 1

### 2.1. Study 1

**Figure 2:**
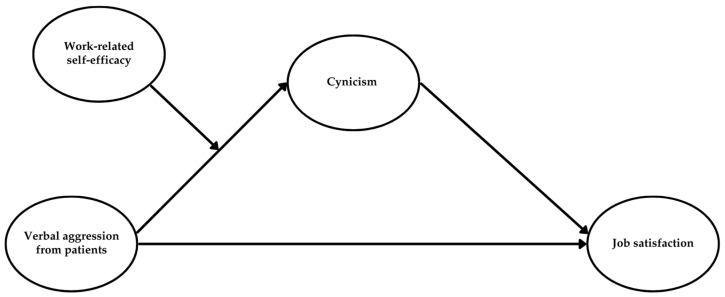
Conceptual model of Study 1: exploring cynicism and work-related self-efficacy.

Study 1 aimed to examine whether verbal aggression from patients would be associated with job satisfaction, as mediated by cynicism and moderated by work-related self-efficacy (Figure 2).

### 2.2. Participants and Procedure- Study 1

This cross-sectional study was conducted in an Italian public hospital in the Lombardy Region from October 2020 to February 2021 during the second wave of COVID-19. The Medical Director, responsible for organizing and coordinating physician services and other professional services within the hospital, commissioned and authorized the study. Staff members were informed about the research via email through the hospital’s intranet system. Ethical approval was granted by the hospital’s Ethical Review Board. The eligibility criteria required participants to be healthcare professionals employed at the hospital who were in contact with patients during the COVID-19 pandemic. Participants also needed to provide informed consent. The research objectives were presented to the professionals by a coordinator and a researcher during shift changes. A total of 201 participants (response rate: 41.44%) completed anonymous self-report paper-and-pencil questionnaires. Four responses were excluded due to incompleteness. The average percentage of missing values ranged from 0% to 1.2%. Little’s MCAR test was non-significant (χ = 84.38, df = 113, and *p* = 0.98), indicating that missing data were completely random. The missing values were replaced using series mean imputation. The questionnaire cover sheet explained the research goals, ensured voluntary participation, and guaranteed response confidentiality. To maintain anonymity, the participants placed completed questionnaires in sealed cardboard boxes. Most respondents were women (78.10%) with an average age of 45.49 years (SD = 10.18, range = 26–61) and 15.39 years of job experience (SD = 12.07, range = 0–41). On average, they spent more than half of their working time in direct contact with the public (M = 52.23%, SD = 36.55).

### 2.3. Measurements—Study 1

Verbal aggression from patients was assessed using the six-item non-physical aggression dimension from the Italian version (Cavallari et al., 2024) of the Hospital Aggressive Behavior Scale (Waschgler et al., 2012). The participants reported the frequency of non-physical aggression (e.g., “Users get angry with me because of delay”) from patients on a 5-point Likert scale (0 = never, 4 = daily).

Cynicism symptoms were evaluated using the five-item dimension from the Italian version of the Maslach Burnout Inventory (Borgogni et al., 2005). The participants reported how frequently they experienced feelings of psychological distance from their work (5 items, e.g., “I have become less enthusiastic about my work”) on a seven-point frequency Likert scale (0 = never, 6 = daily).

Job satisfaction was measured with a single item assessing overall satisfaction (Giorgi et al., 2015; e.g., “How satisfied have you been with your work?”) using a ten-point scale (0 = no satisfaction, 10 = complete satisfaction), where higher scores indicate greater job satisfaction.

Work-related self-efficacy was measured with six items from the Italian version of the Psychological Capital Questionnaire (Alessandri et al., 2015a; Luthans et al., 2007). Participants indicated their perceived capabilities in navigating complex professional situations and managing a range of work-related tasks (e.g., “I feel confident helping to set targets/goals in my work area”) on a seven-point Likert-type scale (1 = “strongly disagree”, 7 = “strongly agree”), with higher scores reflecting greater confidence in their skills and competencies.

## 3. Results—Study 1

### 3.1. Measurement Reliability and Confirmatory Factor Analyses—Study 1

Using IBM SPSS Statistics 25, we initially assessed the psychometric properties of the study scales. Multicollinearity was not a concern, as indicated by variance inflation factors (VIF = 1.19–1.74) and tolerance value range (0.69–0.78), both within acceptable limits. The skewness values ranged from −1.18 to 1.64 and kurtosis from −0.35 to 2.45, both within acceptable limits. All the factor loadings of items on their respective constructs were statistically significant and exceeded the 0.5 cut-off point, suggesting at least a medium correlation with their respective construct (i.e., aggression: 0.74–0.88; cynicism: 0.70–0.84; RESE-NE: 0.79–0.86). Additionally, the composite reliability (CR) coefficients for the study variables ranged from 0.88 to 0.94, and the average variance extracted (AVE) values were above the 0.50 threshold, ranging from 0.60 to 0.71 (i.e., aggression: α = 0.88, CR = 0.91, and AVE = 0.63; cynicism: α = 0.86, CR = 0.90, and AVE = 0.65; RESE-NE: α = 0.91, CR = 0.93, and AVE = 0.70). All the study scales demonstrated satisfactory internal consistency with Cronbach’s alphas ranging from 0.86 to 0.91 (see Table 1).

Using G*Power, we conducted a power analysis for multiple regression with seven predictors, setting the alpha level at 0.05, the power at 0.95, and assuming a medium effect size. The results of this analysis indicated that the required minimum sample size was 153 participants, confirming that our sample size was adequate. However, given the sample size (169) relative to the number of items (17), we employed the parceling technique to maintain an optimal indicator–sample size ratio (Little & Rhemtulla, 2013). Model fit indices can become problematic when the subject–item ratio is below the recommended 10:1 ratio, as in our study. According to Little and Rhemtulla (2013), item parcels improve the sample size–parameter ratio and reduce the likelihood of method effects related to single items. Moreover, item parcels are more reliable as they capture a broader proportion of true score variance, increasing convergence and stability, particularly suitable for models with an unfavorable indicator–sample size ratio (Montani et al., 2020). Following Little and Rhemtulla’s (2013) recommendations, we created three parcels for aggression, cynicism, and self-efficacy by pairing higher-loading items with lower-loading items to balance factor structure.

We then conducted a series of comparative confirmatory factor analyses (see Table 2). The four-factor model demonstrated satisfactory fit (χ^2^ = 48.59, df = 24, *p* = 0.00, RMSEA = 0.08, RMSEA [90% CI] = [0.05, 0.09], SRMR = 0.07, CFI = 0.97, and TLI = 0.95) and outperformed all the alternative models, supporting the distinctiveness of the study variables (see Table 2). Harman’s single-factor test was conducted to detect common method variance. The results showed that the first factor explained 36.41% of the variance without rotation. Thus, no single factor had particularly significant explanatory power. Furthermore, the unmeasured latent method factor accounted for 3.92% of the total variance, which is significantly lower than the 25% average amount of method variance typically observed in self-report research (Podsakoff et al., 2012). This finding suggests that common method variance is unlikely to significantly confound the interpretation of our results.

### 3.2. Hypotheses Testing—Study 1

**Figure 3:**
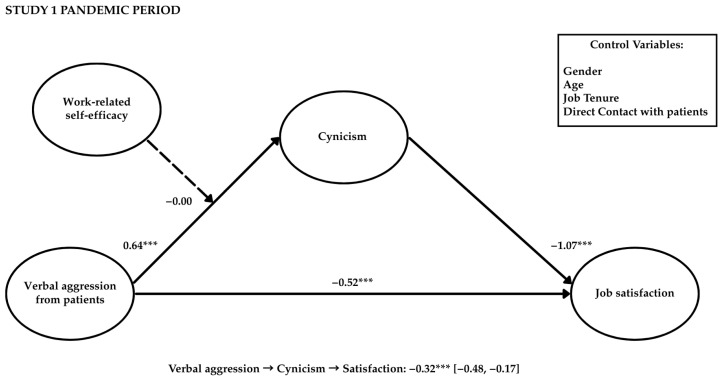
Results of the moderated mediation model tested in Study 1. *** *p* < 0.001.

To examine the hypothesized mediating role of cynicism in the relationship between patient verbal aggression and job satisfaction, we conducted a mediation analysis using the maximum likelihood (ML) method in Mplus Version 8 employing a bootstrapping test with a bias-corrected 95% confidence interval and a resampling procedure of 1000 bootstrap samples. The complementary use of SPSS and Mplus has been endorsed in prior research (e.g., Sommovigo et al., 2018). Specifically, unlike the PROCESS macro in SPSS, Mplus was selected for its advanced capabilities in structural equation modeling, making it particularly well suited for testing complex moderated mediation models. This software allowed us to incorporate latent variables and multiple indicators, ensuring more accurate modeling of the relationships between variables. Additionally, it provides precise estimates of mediation and moderation effects through robust fit indices. Consistent with our hypothesized model, aggression was positively associated with cynicism (β = 0.51, *p* < 0.001, and 95% CI [0.60, 2.21]), which, in turn, was negatively related to job satisfaction (β = −0.63, *p* < 0.001, and 95% CI [−1.44, −0.57]). Although aggression was not directly related to job satisfaction (β = 0.08, ns, and 95% CI [−1.02, 1.30]), cynicism fully mediated the relationship between aggression and job satisfaction (β = −0.32, *p* < 0.001, and 95% CI [−0.48, −0.17]). The results from the moderated mediation model (see Table 3 and Figure 3) demonstrated that the interaction effect was non-significant (β = −0.00, *p* < 0.05, and 95% CI [−0.22, 0.29]), indicating that work-related self-efficacy did not moderate the positive association of aggression with cynicism, allowing employees to remain satisfied with their job. Older healthcare professionals were less likely to be satisfied with their jobs (β = −0.15, *p* < 0.05, and 95% CI [−0.29, −0.00]). No other covariates were statistically significantly related to the study variables. Thus, Hypotheses 1, 2, and 3a were supported, while Hypotheses 4a and 5a were rejected.

## 4. Materials and Methods: Study 2

### 4.1. Study 2

**Figure 4:**
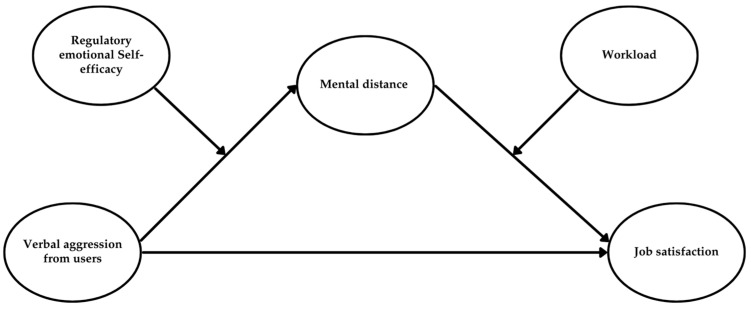
Conceptual model of Study 2: exploring mental distance and regulatory emotional self-efficacy.

Study 2 aimed to replicate the findings from Study 1 by extending the analysis of verbal aggression to users in general (i.e., patients and their relatives) and considering the mediating role of mental distance. Additionally, this study aimed to verify whether RESE-NE could buffer the effects of aggression in terms of mental distance and whether workload could intensify the negative impact of mental distance on job satisfaction (see Figure 4).

### 4.2. Participants and Procedure—Study 2

Our research population comprised healthcare workers from eight healthcare facilities in a Northern Italian region. Although both studies were conducted within the same geographical area, Study 2 was conducted in different healthcare settings during the post-pandemic period (in contrast to the pandemic period in Study 1). Additionally, Study 2 did not include the specific hospital where Study 1 was conducted. This methodological distinction was intentional, allowing us to assess potential variations in the impact of verbal aggression across different healthcare settings and periods. This approach enhances the generalizability of our findings and provides a broader understanding of the phenomenon under investigation. This study, conducted between May and June 2024, adhered to the ethical standards of the Italian National Psychological Association and received approval from the Ethical Committee of the University of Pavia. The researchers prepared a formal and detailed communication outlining the project objectives and operational methods. This communication was sent to the management of various departments and subsequently cascaded down to all the employees through their direct supervisors. The participants were assured of the anonymity of their responses, gave written informed consent, and completed online questionnaires, which required approximately 15 min to complete. A total of 1660 healthcare workers (response rate: 28.38%) participated in the survey. We excluded 12 cases due to incomplete answers (less than 60% of the survey), reducing the sample size to 1648. Following previous studies (e.g., Milani et al., 2024; Bernuzzi et al., 2022), multivariate outliers were identified using the Mahalanobis distance criterion with a significance threshold of *p* < 0.001, a widely accepted method for detecting cases that deviate significantly from the overall data distribution. The presence of multivariate outliers can disproportionately influence parameter estimates and compromise the validity of statistical inferences (Tabachnick & Fidell, 2013). To enhance the robustness of the analyses and mitigate potential biases, we removed 206 cases identified as multivariate outliers, resulting in a final sample of 1442 respondents. The average percentage of missing values for continuous variables ranged from 0% to 2.1%. The results of Little’s MCAR test were statistically non-significant (χ^2^ = 11.74, df = 18, and *p* = 0.86), indicating that the data were completely missing at random. We then replaced missing entries with the average of the corresponding series. Most respondents were women (77.40%) with an average age of 48.22 years (SD = 10.96, range = 21–78) and 21.36 years of job experience (SD = 12.01, range = 0–56). On average, they spent most of their time in direct contact with the public (M = 66.71%, SD = 32.13).

### 4.3. Measurements—Study 2

As in Study 1, the participants were invited to complete the non-physical aggression dimension of the Italian version of the Hospital Aggressive Behavior Scale (Cavallari et al., 2024) and a single item assessing overall job satisfaction (Giorgi et al., 2015). However, they were instructed to consider aggression not only from patients but also from visitors, recognizing that both groups can be potential sources of verbal aggression in the post-pandemic context. Moreover, unlike Study 1, we measured mental distance with the recent Burnout Assessment Tool and RESE-NE.

Mental distance was assessed using the Italian version of the short form of the Burnout Assessment Tool (Mazzetti et al., 2022). The participants responded to five items (e.g., “I’m cynical about what my work means to others”) reflecting their experiences at work, such as struggling to find enthusiasm, operating on autopilot, displaying indifference towards job responsibilities, and harboring cynical views about the significance of their work using a five-point Likert-type scale (1 = “never”, 5 = “always”).

Regulatory emotional self-efficacy beliefs in the workplace were evaluated using six items from the regulatory emotional self-efficacy scale, specifically adapted for organizational contexts (Alessandri et al., 2015b). The participants were asked to rate their perceived ability to manage negative emotional states triggered by work-related events (e.g., “I can remain calm during stressful situations and job-related frustrations”) on a five-point Likert scale (1 = “strongly disagree”, 5 = “strongly agree”).

Workload was measured using the six-item subscale from the Italian version (Borgogni et al., 2005) of the Organizational Check-up System (Maslach & Leiter, 2005). The participants rated their agreement with statements related to their perceptions of workload, including time constraints and work intensity (e.g., “I do not have time to do the work that needs to be done”) on a seven-point Likert-type scale (1 = strongly disagree; 7 = strongly agree).

## 5. Results—Study 2

### 5.1. Measurement Reliability and Confirmatory Factor Analyses—Study 2

Using IBM SPSS Statistics 25, we initially assessed the psychometric properties of the study scales. Multicollinearity was not a concern, as indicated by the VIF values ranging from 1.09 to 1.56 and tolerance values between 0.64 and 0.92, well below the recommended threshold of 10. The skewness values ranged from −0.94 to 1.31 and kurtosis from −0.13 to 1.54, both within acceptable limits. All the factor loadings of items on their respective constructs were statistically significant and exceeded the 0.5 cut-off point, suggesting at least a medium correlation with their respective construct (i.e., aggression: 0.71–0.86; mental distance: 0.67–0.86; RESE-NE: 0.81–0.86; workload: 54–86). Additionally, the CR coefficients for the study variables ranged from 0.88 to 0.94, and the AVE values were above the 0.50 threshold (i.e., aggression: α = 0.88, CR = 0.91, and AVE = 0.64; mental distance α = 0.80, CR = 0.88, and AVE = 0.59; RESE-NE: α = 0.92, CR = 0.94, and AVE = 0.71; workload: α = 0.78, CR = 0.85, and AVE = 0.51). All the study scales demonstrated satisfactory internal consistency with Cronbach’s alphas ranging from 0.78 to 0.92 (see Table 4). The subject–item ratio met the recommended 10:1 ratio, requiring a minimum of 270 participants for 24 items and four covariates, enabling the implementation of comprehensive latent variable models. The fit indices of the four-factor model were satisfactory (χ^2^ = 1499.49, df = 224, *p* = 0.00, RMSEA = 0.07, RMSEA [90% CI] = [0.06, 0.07], SRMR = 0.05, CFI = 0.91, TLI = 0.90) and outperformed all alternative models, supporting the distinctiveness of the study variables (see Table 5).

Harman’s single-factor test was conducted to detect common method variance. The results showed that the first factor explained 28.88% of the variance without rotation. Thus, no single factor accounted for a disproportionately large share of the variance. Furthermore, the unmeasured latent method factor accounted for 9.00% of the total variance, which is significantly lower than the 25% average amount of method variance typically observed in self-report research (Podsakoff et al., 2012). Thus, common method variance is unlikely to confound the interpretation of our results.

### 5.2. Hypotheses Testing

To examine the hypothesized mediating role of mental distance in the relationship between aggression and job satisfaction, we conducted a mediation analysis using the ML method in Mplus Version 8. We employed a bootstrapping test with a bias-corrected 95% confidence interval and a resampling procedure of 1000 bootstrap samples. Consistent with our hypothesized model, aggression was positively associated with mental distance (β = 0.32, *p* < 0.001, and 95% CI [0.23, 0.43]), which, in turn, was negatively related to job satisfaction (β = −0.55, *p* < 0.001, and 95% CI [−1.68, −1.29]). Aggression was not directly related to job satisfaction (β = 0.04, *p* = 0.23, and 95% CI [−0.34, 0.07]). Mental distance fully mediated the relationship between aggression and job satisfaction (β = −0.18, *p* < 0.001, and 95% CI [−0.24, −0.13]). The results remained invariant regardless of whether the covariates were included.

**Figure 5:**
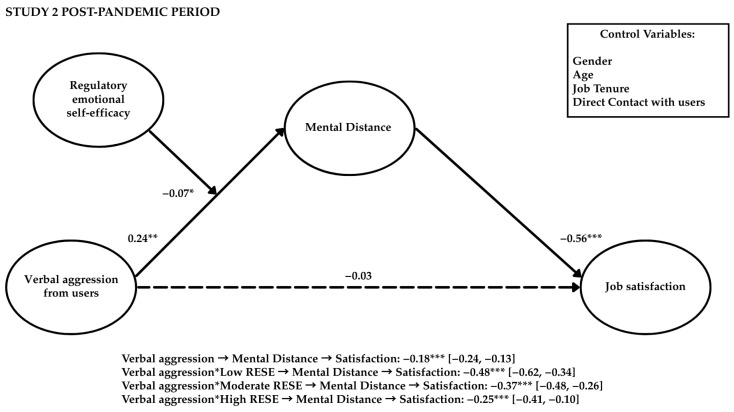
Results of the moderated mediation model tested in Study 2 (in post-pandemic times). * *p* < 0.05, ** *p* < 0.01, and *** *p* < 0.001.

The results from the moderated mediation model (see Table 6; Figure 5) demonstrated that RESE-NE moderated the relationship between aggression and mental distance. The interaction effect was negative (β = −0.07, *p* < 0.05, and 95% CI [−0.13, −0.01]), indicating that RESE-NE mitigated the positive impact of aggression on mental distance, helping employees maintain job satisfaction. The moderated mediation index was statistically significant (β = 0.11, *p* < 0.05, and 95% CI [0.02, 0.21]).

Specifically, healthcare professionals with low (β = −0.48, *p* < 0.001, and 95% CI [−0.62, −0.34]) and moderate (β = −0.37, *p* < 0.001, and 95% CI [−0.48, −0.26]) levels of RESE-NE were more susceptible to experiencing mental distance and then job dissatisfaction when facing user aggression. In contrast, for healthcare professionals with high RESE-NE, the indirect effect was weaker (β = −0.25, *p* < 0.001, and 95% CI [−0.41, −0.10]). Having less professional experience and spending more time in direct contact with the public (β = 0.15, *p* < 0.001, and 95% CI [0.09, 0.30]) were positively associated with a higher likelihood of exposure to user aggression. Women were less likely to develop mental distance (β = −0.09, *p* < 0.01, and 95% CI [−0.14, −0.03]) and experience job dissatisfaction (β = −0.07, *p* < 0.01, and 95% CI [−0.12, −0.02]). Healthcare professionals with longer job tenure were more likely to develop mental distance (β = 0.19, *p* < 0.001, and 95% CI [0.08, 0.30]).

Next, we conducted a second stage moderated mediation model with workload as a moderator of the mental distance–job satisfaction link. Both the interaction term (β = −0.13, *p* < 0.05, and 95% CI [−0.24, −0.11]) and the index of moderated mediation (β = −0.04, *p* < 0.01, and 95% CI [−0.07, −0.02]) were statistically significant. The results indicate that healthcare professionals subjected to user verbal aggression were more likely to respond with mental distancing and, then, experience job dissatisfaction when workload was high (β = −0.34, *p* < 0.01, and 95% CI [−0.48, −0.20]) compared to when workload was moderate (β = −0.30, *p* < 0.01, and 95% CI [−0.43, −0.17]) or low (β = −0.26, *p* < 0.01, and 95% CI [−0.40, −0.13]).

**Figure 6:**
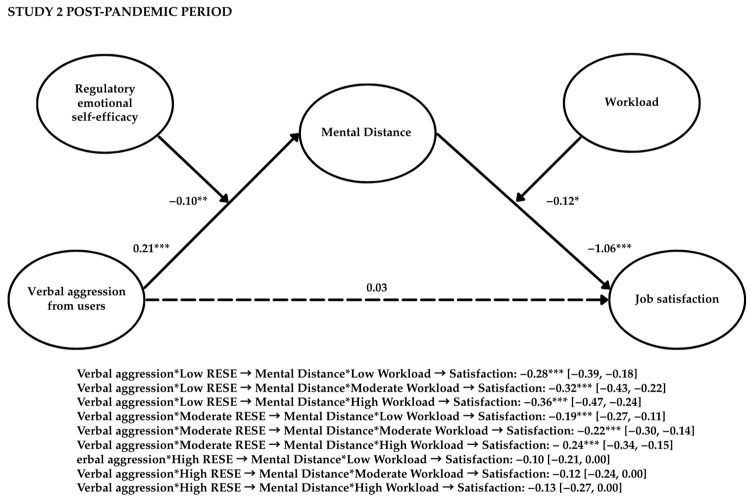
Results of the moderated mediation model having regulatory self-efficacy in managing negative emotions and workload as moderators. * *p* < 0.05, ** *p* < 0.01, and *** *p* < 0.001.

Table 7 and Figure 6 present the findings from the moderated mediation model, examining the simultaneous moderating effects of RESE-NE and workload. The results indicated that higher levels of RESE-NE mitigated the negative relationship between user verbal aggression and mental distance. At the same time, an increased workload exacerbated the detrimental impact of mental distance on job satisfaction. Interaction terms revealed nuanced dynamics: the impact of mental distance on job satisfaction was dependent on both RESE-NE and workload levels. The most adverse outcomes were observed in healthcare professionals with low RESE-NE and high workload (β = −0.36, *p* < 0.001, and 95% CI [−0.47, −0.24]), followed by those with low RESE-NE and moderate (β = −0.32, *p* < 0.001, and 95% CI [−0.43, −0.22]) or low (β = −0.28, *p* < 0.001, and 95% CI [−0.39, −0.18]) workloads. In contrast, high RESE-NE appeared to buffer against the detrimental effects of aggression, regardless of workload. The conditional indirect effects of mental distance on job satisfaction were not statistically significant at low (β = −0.10, ns, and 95% CI [−0.21, 0.00]), moderate (β = −0.12, ns, and 95% CI [−0.24, 0.00]), or high (β = −0.13, ns, and 95% CI [−0.27, 0.00]) workload levels when healthcare professionals possessed high RESE-NE. These findings underscore the importance of RESE-NE as a key personal resource, safeguarding healthcare workers from the adverse effects of user verbal aggression, independent of workload levels. Hypothesis 1 was not supported, whereas Hypotheses 2b, 3b, 4b, 5b, 6, 7, and 8 were supported.

## 6. Discussion

This research, consisting of two studies, advances the workplace aggression literature by unveiling the psychological withdrawal mechanisms that explain how verbal aggression from users is negatively associated with job satisfaction among healthcare professionals. By identifying both the personal (i.e., RESE-NE) and contextual (i.e., workload) factors that shape this relationship, the studies provide a nuanced understanding of how and when healthcare professionals react to non-physical aggression. Specifically, cynicism was evaluated during the pandemic, while mental distance was assessed in the post-pandemic period. Notably, while the direct association between aggression and job satisfaction was significant during the pandemic, it was not in post-pandemic times. This change may be attributed to the extraordinary stressors experienced during the pandemic—such as exhausting shifts, heightened fears of infection, uncertainty, restrictive protocols, shortage of personnel and protective equipment, and increased work-family conflict—which probably intensified the perceived threat of verbal aggression, and magnified its association with job satisfaction (Sommovigo et al., 2022b). Conversely, the post-pandemic period likely provided healthcare workers with opportunities for recovery, and a more stable environment, which reduced the direct harmful effects of user verbal aggression (Doleman et al., 2023). Ultimately, this study contributes to an increasing body of research that identifies non-physical aggression as a significant job demand. It highlights the role of such aggression in initiating a cyclical process of energy depletion, and cynicism (Goussinsky, 2024; Goussinsky & Livne, 2019), which undermines job satisfaction (Toscanelli et al., 2023).

By demonstrating that cynicism and mental distancing are the key pathways through which verbal aggression is negatively related to job satisfaction in both emergency and post-pandemic contexts, this study deepens our understanding of how aggression shapes healthcare workers’ attitudes. Previous workplace aggression research has predominantly focused on burnout or emotional exhaustion without differentiating among its specific dimensions (Sommovigo et al., 2022a). The findings clarify that both emotional withdrawal (e.g., emotional detachment from users and disillusionment toward work, typical of cynicism; Montgomery & Maslach, 2019) and cognitive withdrawal (e.g., mental detachment from work tasks and the work environment, characteristic of mental distancing; Schaufeli & De Witte, 2023) significantly explain how healthcare professionals experience job dissatisfaction. This disengagement serves as a coping response to resource depletion triggered by verbal aggression (Cao et al., 2023; Vidal-Alves et al., 2022). These forms of psychological withdrawal act as short-term resource-conservation strategies that, while helping professionals protect their remaining resources, ultimately result in reduced emotional investment and job satisfaction (Hodkinson et al., 2022). This aligns with the less-explored desperation principle within the Conservation of Resources theory (Hobfoll et al., 2023), which posits that resource-depleted individuals may resort to protective mechanisms, such as psychological withdrawal, to prevent further resource losses (Pina et al., 2022).

This study enhances the existing literature on coping strategies in response to verbal aggression in healthcare settings. While previous research has primarily focused on general coping mechanisms—such as problem-focused strategies (e.g., seeking support and skill development) and emotion-focused strategies (e.g., emotional regulation and cognitive reappraisal) to manage workplace stressors (Gabriel et al., 2023)—our findings emphasize psychological withdrawal as a distinct self-protective response. This disengagement allows individuals to conserve psychological resources when faced with ongoing verbal aggression (Vidal-Alves et al., 2022; Cao et al., 2023; Pina et al., 2022). In high-stress environments like healthcare, where exposure to verbal aggression is common and often unavoidable, strategies such as mental distancing and cynicism can serve as adaptive short-term responses. These strategies can help professionals reduce emotional strain and maintain functionality (Toscanelli et al., 2023). However, although psychological withdrawal may offer temporary relief, prolonged reliance on such disengagement strategies can have harmful long-term effects (Leiter & Maslach, 2024; Pina et al., 2022; Vidal-Alves et al., 2022; Cao et al., 2023). Continuous detachment from work-related interactions is associated with increased emotional exhaustion, decreased organizational commitment, and lower job satisfaction (Cao et al., 2023; Hodkinson et al., 2022). Moreover, this withdrawal can compromise the quality of patient care, as healthcare professionals who mentally disengage from their roles risk losing empathy, becoming less attentive to patient needs, and increasing the likelihood of medical errors (Pina et al., 2022; Vidal-Alves et al., 2022; Cao et al., 2023; Hodkinson et al., 2022). Thus, our findings enhance the understanding of how workplace aggression—especially verbal aggression from patients and visitors—is negatively related to healthcare workers’ job satisfaction through the mediating role of psychological withdrawal.

This study contributes to self-efficacy research by showing that work-related self-efficacy among healthcare workers does not mitigate the adverse effects of verbal aggression from patients. This suggests that such aggression uniformly affects workers negatively, regardless of their confidence in performing job tasks or meeting performance expectations (Luthans et al., 2007). A possible explanation is that work-related self-efficacy primarily focuses on task execution, fostering confidence in technical and procedural abilities but not adequately preparing workers for the emotional challenges that arise when interacting with verbally aggressive users (Luthans et al., 2007). Verbal aggression triggers intense emotional reactions—such as anger, anxiety, and stress—that may be beyond the protective scope of self-efficacy focused on task performance (Gilardi et al., 2020). While self-efficacy can enhance resilience in managing job functions, it does not adequately shield healthcare workers from emotionally distressing encounters with aggressive users. Thus, work-related self-efficacy appears insufficient in mitigating the psychological strain of verbal aggression, as the primary challenges in these encounters stem from emotional rather than task-related demands. The Conservation of Resources theory provides a theoretical basis for understanding the moderating role of RESE-NE (Hobfoll et al., 2015). Verbal aggression in the workplace constitutes a significant psychosocial stressor that demands immediate cognitive and emotional regulation, thus depleting psychological resources (Koopmann et al., 2015; Hobfoll et al., 2023). In demanding environments like healthcare, professionals must manage frequent instances of verbal aggression, while adhering to emotional display rules—organizational norms that require the suppression of negative emotions to maintain professional composure (Grandey & Melloy, 2017). This suppression is crucial for preventing escalation and preserving the care relationship (Gilardi et al., 2020). Successfully navigating these situations necessitates significant self-regulatory resources, including sustained attention, emotional suppression, and cognitive control (Sliter et al., 2010; Sommovigo et al., 2024). Individuals with high RESE-NE experience a protective effect because they have greater confidence in their ability to regulate negative emotional states. This confidence allows them to use more adaptive emotion regulation strategies, minimizing resource depletion and preserving psychological energy for work-related engagement (Bandura, 2023; Sommovigo et al., 2024). By viewing aggressive interactions as manageable rather than threatening, they activate effective coping mechanisms, minimizing emotional spillover and protecting job attitudes (Madrid et al., 2020; Grandey & Melloy, 2017). Thus, RESE-NE can serve as a vital personal resource that shields employees from the harmful effects of interpersonal mistreatment on their well-being and job satisfaction. As such, while work-related self-efficacy may assist in managing job tasks, it is inadequate for coping with the emotional strain of verbal aggression.

The COVID-19 pandemic significantly increased the emotional strain on healthcare professionals, introducing unprecedented psychological challenges (Maffoni et al., 2022). Alongside managing medical and logistical demands, healthcare workers faced ethical dilemmas and the emotional distress of patients who were grappling with fear, grief, and uncertainty in highly charged environments (Koontalay et al., 2021; Maffoni et al., 2022). These compounded stressors placed extraordinary pressure on emotional regulation, surpassing the challenges experienced prior to the pandemic (Sexton et al., 2022). Traditional work-related self-efficacy, focusing on technical skills and task performance, has proved insufficient in addressing this prolonged emotional toll. While it can support procedural and cognitive demands, it does not alleviate the lasting psychological burden. In contrast, RESE-NE has emerged as a crucial resource for fostering resilience and maintaining well-being in high-stress settings. This shift from work-related self-efficacy to RESE-NE reflects broader changes in healthcare, moving from crisis response to long-term adaptation. During the pandemic, professionals faced extreme occupational stressors, including extended shifts, strict safety protocols, and fear of infection (Maffoni et al., 2022). Initially, confidence in technical skills and problem solving was paramount. However, as operational disruptions began to ease, emotional fatigue and compassion depletion became the dominant concerns (Lluch et al., 2022; Sexton et al., 2022). In this new landscape, a higher level of RESE-NE empowers professionals to sustain their psychological well-being and maintain their job satisfaction, making it a particularly relevant form of self-efficacy in the post-pandemic era.

Integrating the Conservation of Resources theory (Hobfoll et al., 2023) with the Social Cognitive theory (Bandura, 2023), RESE-NE emerges as a personal coping resource that enables individuals to respond to emotionally charged situations with lower psychological stress and attenuated neuroendocrine responses (Sommovigo et al., 2023). This capacity arises from a heightened threshold for perceiving threats, allowing those with high RESE-NE to interpret verbal aggression as less personally threatening, thereby experiencing reduced distress and greater resilience in their responses (Bandura, 2023; Sommovigo et al., 2023). This enables them to better preserve emotional resources and adopt resource-building strategies that protect against potential resource losses (Hobfoll et al., 2015; Kwak et al., 2020). As a result, they can sustain the emotional energy essential for investing emotionally in their roles and remaining satisfied with their jobs (Hobfoll et al., 2023; Sommovigo et al., 2024; Madrid et al., 2020). This study advances the self-efficacy literature by introducing RESE-NE and shifting the focus from traditional emotion regulation strategies to personal beliefs about managing negative emotions. While most studies examine specific self-regulation techniques (e.g., deep acting; Goussinsky & Livne, 2019) for handling interactions with misbehaving users, RESE-NE highlights the protective role of underlying confidence in one’s capacity to handle these emotions effectively, preserving emotional resources in high-stress environments. This perspective adds a crucial layer to the research on workplace aggression, showing that RESE-NE can buffer healthcare professionals from the effects of verbal aggression, safeguarding against dissatisfaction.

This study also examines the combined impact of RESE-NE and workload on job satisfaction. The findings reveal that the negative indirect effect of user aggression on job satisfaction, mediated by mental distance, is especially severe for healthcare workers with low RESE-NE who face high workloads. Conversely, those with high RESE-NE demonstrate resilience to these adverse effects, effectively counteracting the energy-depleting effects of user verbal aggression regardless of workload levels. Professionals with high RESE maintain a positive, emotionally engaged attitude toward their roles, even when interacting with verbally aggressive users and managing heavy workloads. These results underscore the importance of both RESE-NE and workload in shaping healthcare professionals’ responses to aggression, offering a nuanced understanding of how these factors interact to shape job satisfaction in high-stress healthcare settings. Unlike previous studies that have explored the joint moderating effects of self-efficacy and contextual resources (e.g., job autonomy, Goussinsky, 2024; co-worker support Goussinsky, 2020), this research highlights how an internal resource—RESE-NE—can safeguard against the harmful effects of user aggression, even under resource-poor conditions, such as excessive workload. This supports the assertion of the Conservation of Resources theory that personal resources become especially valuable under challenging conditions, offering critical protection in high-demand environments (Hobfoll et al., 2018). Moreover, this study adds to the emotional labor literature by elucidating how healthcare professionals manage the emotional burden associated with user-initiated verbal aggression. Emotional labor (i.e., the regulation of emotions to fulfill job demands) has been widely studied in service professions. A significant focus of this research has been on two strategies: surface acting (i.e., which involves modifying outward expressions) and deep acting (i.e., which entails altering internal feelings; Grandey & Melloy, 2017). However, this body of work identifies psychological withdrawal mechanisms as alternative coping strategies. The findings extend prior emotional labor research by underscoring emotional and psychological detachment as a distinct withdrawal response to emotionally taxing interactions. Traditionally, emotional regulation has been viewed as an effortful process aimed at sustaining positive workplace interactions (Grandey & Melloy, 2017). Nonetheless, this study reveals that employees who frequently experience verbal aggression may opt for disengagement rather than traditional emotional labor strategies, especially when they feel they lack emotional coping resources (i.e., low RESE-NE) and are in resource-depleted environments (i.e., high workload). This evidence underscores the importance of personal affective resources in emotionally demanding professions.

Higher levels of public interaction correlate with an increased likelihood of experiencing verbal aggression, which is consistent with previous research (Sommovigo et al., 2022b). Additionally, healthcare workers with less professional experience often report greater job dissatisfaction, likely due to underdeveloped emotional regulation skills and coping strategies early in their careers (Cavallari et al., 2024). In contrast, female healthcare workers tend to report lower levels of mental distance and higher job satisfaction, which may reflect gender-based strengths in empathy and emotional support (Milfont & Sibley, 2016). On the other hand, healthcare professionals with longer job tenures display higher mental distance, suggesting that they may adopt this strategy over time to cope with ongoing stressors, potentially experiencing feelings of disillusionment. Finally, in line with the findings from other studies (Raab, 2019), older healthcare workers report lower job satisfaction. This may be attributed to factors such as limited autonomy—in which their desire for greater control over work schedules is restricted by rigid institutional structures—and a lack of recognition, as they may feel undervalued compared to their younger counterparts (Rice et al., 2021).

### 6.1. Limitations and Future Directions

This study has limitations that suggest promising directions for future research. First, it relied on cross-sectional, self-reported data from a sample of healthcare workers in a single region of Northern Italy. This approach may introduce common method variance and limit causal inference. Nonetheless, following methodological guidance (Podsakoff et al., 2012), we controlled for common method bias and found it unlikely to pose a significant issue in our study. Although our data were collected from several healthcare facilities in both pre- and post-pandemic periods, future longitudinal studies with larger, more diverse samples of healthcare professionals from various regions of Italy and other countries could enhance the generalizability of the findings. Future studies could integrate multi-source self-reports with objective measures (e.g., recorded critical events) and qualitative data (e.g., interviews with victims).

Moreover, we must consider the temporal context of Study 1, which was conducted during the COVID-19 pandemic. This study captured the effects of verbal aggression in a high-stress environment characterized by increased emotional strain and restricted hospital access. While this provided a unique opportunity to examine workplace aggression under crisis conditions, it also implies that the findings may not be fully generalizable to other periods, such as pre-pandemic or long-term post-pandemic healthcare settings. Although Study 2 aimed to replicate and extend the findings in a post-pandemic context, we cannot discount the possibility that the results might have differed if Study 1 had been conducted before or after the pandemic. Future research should employ longitudinal designs or compare pre-pandemic and post-pandemic datasets to assess how the impact of verbal aggression evolves over time. While both studies examined self-efficacy as a moderator, we used work-related self-efficacy in Study 1 and RESE-NE in Study 2 to reflect the distinct challenges posed by the pandemic and post-pandemic periods. This difference in measurement, though intentional, limits direct comparability between the two studies. Although RESE-NE emerged as a more relevant resource in post-pandemic times—where emotional labor became a primary challenge—future research should explore whether both types of self-efficacy interact or complement each other over time. Examining both work-related self-efficacy and RESE-NE within the same study could provide a more nuanced understanding of how healthcare workers’ coping mechanisms shift across different workplace conditions. While our findings align with theoretical expectations and prior research, a longitudinal design would allow for stronger conclusions about the directionality of relationships, particularly regarding how verbal aggression influences job satisfaction and psychological withdrawal over time. Future research should explore temporal patterns in workplace aggression, including whether prolonged exposure to verbal aggression leads to cumulative emotional exhaustion or whether certain coping strategies mitigate long-term effects. Additionally, future research should investigate whether daily experiences of verbal aggression led to fluctuations in self-efficacy, which may, in turn, impact job satisfaction. Thus, recent studies suggest that personal resources can vary in response to daily social stressors (Sommovigo et al., 2024).

Despite the methodological reasoning behind the differences in sample sizes—specifically, using a two-step approach where a smaller sample was used for preliminary testing and a larger sample for replication and extension—we acknowledge that the imbalance between Study 1 and Study 2 poses certain limitations. Thus, the smaller sample in Study 1 may restrict the generalizability of its findings, as the limitations in statistical power could impact the reliability of the observed relationships. Additionally, the differences in data collection periods (during the pandemic versus post-pandemic) introduce potential contextual variability that may have influenced how the participants perceived verbal aggression, psychological withdrawal, and job satisfaction. To address these concerns, we collected data from the same geographical region and ensured demographic comparability across the samples (e.g., gender distribution). Future research could build on these findings by using more balanced sample sizes to enhance external validity or by adopting longitudinal designs to capture the dynamic nature of these relationships over time.

Future studies could also incorporate additional variables to deepen the understanding of the mechanisms at play (e.g., resource consumption processes), alongside personal and contextual factors. These factors may act as boundary conditions that jointly shape healthcare professionals’ responses to verbal aggression from users. In this context, exploring how a balanced integration of job demands and resources can strengthen the professional’s resilience in high-stress environments would be especially valuable. Further research could extend our findings by distinguishing between different types of verbal aggression (e.g., face-to-face versus online), and various forms of workplace aggression (e.g., verbal versus physical) and their sources (e.g., internal versus external). This approach could help clarify their distinct or cumulative effects on physiological withdrawal and job satisfaction. Additionally, future studies could examine the contagion effects of verbal aggression on bystanders and explore how such aggression in the workplace might impact family life, contributing to a work-to-family spillover effect.

Future studies could also explore how RESE-NE interacts with different emotional self-regulation strategies (e.g., surface acting versus deep acting) to effectively respond to external demands and emotional stimuli in the workplace, particularly in incidents of workplace aggression. Additionally, more research is needed to understand how the application of momentary emotional self-regulation strategies in response to daily occurrences of user-initiated verbal aggression may affect daily job satisfaction depending on individual levels of trait RESE-NE.

Another limitation of this study is that we did not systematically analyze differences based on occupational roles. Although our sample included a diverse range of healthcare professionals, the distribution of occupations was highly imbalanced across both studies, with the participants coming from multiple facilities and departments. This diversity enhances the generalizability of our findings but also restricts our ability to draw occupation-specific conclusions. Given that various healthcare roles may involve differing levels of exposure to verbal aggression, distinct coping mechanisms, and varying degrees of emotional labor, future research should adopt a more targeted approach to ensure a balanced representation across professions and employ analytical techniques capable of capturing role-specific variations.

A further limitation pertains to the characterization of the post-pandemic period. While Study 2 was conducted after the acute phase of the COVID-19 crisis, it does not necessarily reflect a complete return to the normal conditions that existed prior to the pandemic. Healthcare environments have experienced lasting structural and psychological changes, including accumulated emotional strain, ongoing staff shortages, and shifts in patient behavior. These factors may continue to influence the experiences of healthcare professionals. Although we differentiate the post-pandemic context from the exceptional crisis state of the pandemic, we do not assume that the psychological dynamics observed in Study 2 reflect those of a true baseline “normal” period. Future research could benefit from longitudinal designs or comparisons with pre-pandemic baseline data to better understand how workplace dynamics and coping mechanisms have changed over time.

Lastly, due to the voluntary nature of participation in this study, we must acknowledge the potential for selection bias. Our sample may be impacted by the “healthy worker effect” (Sommovigo et al., 2022b), which could lead to an underestimation of psychological withdrawal responses. It seems likely that the participants predominantly included individuals who were psychologically healthier and able to remain actively engaged in their roles. In contrast, those experiencing significant emotional distress or detachment may have been underrepresented, possibly due to work absences or reluctance to participate. Furthermore, we did not account for the history of psychiatric treatment, which could have offered additional insights into individual differences in coping mechanisms and susceptibility to emotional strain. Pre-existing mental health conditions may influence both psychological withdrawal responses and RESE-NE, which could affect our findings. Future research should consider including this variable to gain a better understanding of the pre-existing vulnerabilities that shape how healthcare professionals react to verbal aggression. To reduce selection bias in future studies, providing incentives for participation could help ensure broader representation among all hospital staff, including those experiencing higher levels of emotional strain. This strategy would enhance the diversity of the sample and offer a more comprehensive view of the workforce.

### 6.2. Practical Implications

From a practical standpoint, healthcare organizations should prioritize strategies to prevent verbal aggression from users. Implementing a clear, organization-wide zero-tolerance policy for verbal aggression is essential. This policy should establish and communicate specific guidelines on acceptable and unacceptable behaviors (e.g., via visible anti-aggression signage and reinforced communications). Developing a systematic reporting system to document incidents of verbal aggression is also critical, as it enables organizations to identify patterns, understand triggers, and develop targeted prevention strategies that address underlying causes of aggression. These records can also serve as case studies in training programs, allowing staff to examine real-world situations and engage in experiential, evidence-based learning. Interactive training methods, such as simulations, scenario-based exercises, and role-playing, can effectively help healthcare workers (especially those in constant contact with the public) learn de-escalation techniques. Training programs should also cover essential skills in conflict resolution, stress management, and emotional regulation, enabling staff to diffuse potentially aggressive situations. Emotion regulation training can focus on various techniques, including mindfulness-based stress reduction, relaxation techniques, and cognitive restructuring. These approaches can help employees reframe hostile encounters and process their emotions more constructively (Montani et al., 2023). For example, mindfulness exercises allow healthcare workers to become more aware of their emotional responses in real time, enabling them to regulate their reactions during stressful situations (Baby et al., 2019). Additionally, deep breathing exercises can activate the parasympathetic nervous system, promoting calmness in intense situations (Catapano et al., 2023). Cognitive restructuring can help employees reframe negative thoughts, helping them manage aggressive encounters more effectively as manageable challenges (Montani et al., 2023). By equipping healthcare workers (especially those with less professional experience) with these skills, organizations can enhance employees’ self-efficacy in managing difficult interactions and boost their overall resilience.

To prevent healthcare professionals from developing psychological withdrawal in response to verbal aggression, healthcare managers should create “environmental conditions that foster, enrich, and protect” employees’ resources—a concept referred to as “caravan passageways” (Hobfoll et al., 2018). This approach emphasizes the need for organizational structures that enable workers to draw on shared resources to manage challenging interactions, thus preserving their emotional reserves.

At the team level, interventions can strengthen social bonds and cultivate the supportive networks needed to handle aggressive behaviors from users. Supervisors should promote this supportive culture by organizing regular group debriefing sessions, where team members can openly share both their emotional struggles and successes. These sessions not only help staff process their experiences but also reinforce their awareness of their strengths and resources, building collective resilience and promoting recovery from demanding encounters. Moreover, supervisors should maintain consistent communication and mentoring with their staff, using these opportunities to discuss difficult interactions and collaboratively explore solutions for addressing user concerns. This personalized support enables healthcare professionals to feel heard and prepared to tackle future challenges, reducing the risk of psychological withdrawal. Healthcare managers should also implement comprehensive workload management practices to support staff. Key strategies include ensuring adequate staffing levels, enabling flexible scheduling, and rotating high-stress assignments to balance demands. Establishing clear guidelines for task prioritization can help focus team efforts on critical clinical tasks, making workloads more manageable. Advanced systems, such as patient acuity models that categorize patients according to their care needs, can facilitate real-time personnel allocation, ensuring resources align with patient demands. Integrating technology, including electronic health records and automated scheduling systems, can further improve task efficiency. Routinely monitoring workloads, through feedback and data analysis, is essential for maintaining balanced work environments. Additionally, providing mental health resources—such as psychological support services—can help staff handle the emotional challenges associated with heavy workloads and user interactions, including incidents of aggression. By nurturing a workplace culture that prioritizes mutual support, healthcare organizations can mitigate the adverse effects of aggression, helping staff maintain satisfaction in their roles.

## 7. Conclusions

This two-study research sheds light on how and under what conditions verbal aggression from users is related to psychological withdrawal and job dissatisfaction among healthcare workers. This research represents a significant contribution, as it is the first to show that low RESE-NE, combined with excessive workload, creates a dual vulnerability, intensifying the harmful effects of verbal aggression. The findings offer valuable, practical insights for implementing both individual-focused and organizational strategies to protect healthcare professionals from emotional and mental detachment, as well as job dissatisfaction. We hope this study will inspire further longitudinal and intensive daily research into the mediating and moderating factors shaping the impact of verbal aggression. Ultimately, we hope this research will guide the development of evidence-based interventions to safeguard the well-being of those dedicated to patient care.

## Figures and Tables

**Table 1:** Descriptive statistics and correlations among variables in Study 1.

STUDY 1—PANDEMIC TIMES											
	M	SD	Sk.	Kur.	(1)	(2)	(3)	(4)	(5)	(6)	(7)
1. Aggr.	0.61	0.72	1.61	2.45	**0.88**						
2. Cynis.	1.50	1.42	0.90	−0.06	0.45 **	**0.86**					
3. Sat.	6.51	2.05	-	-	−0.19 **	−0.54 **	**-**				
4. SE	5.05	1.05	−0.30	−0.35	−0.25 **	−0.34 **	0.33 **	**0.91**			
5. Sex	-	-	-	-	−0.05	−0.12	0.01	−0.13	**-**		
6. Age	45.49	10.18	-	-	0.07	0.04	−0.14	−0.08	0.08	**-**	
7. Ten.	15.39	12.07	-	-	0.04	0.07	−0.14	−0.21 **	0.10	0.51 **	**-**
8. Contact	52.23	36.55	-	-	0.24 **	0.07	−0.07	−0.14	0.11	0.01	0.10
9. Wload	-	-	-	-	-	-	-	-	-	-	-

Note. Boldfaced numbers on the diagonal represent Cronbach’s alpha; Aggr. = patient verbal aggression, Cynis. = cynicism, Sat. = job satisfaction, SE. = work self-efficacy, M = mean, SD = standard deviation, Sk. = skewness, Kur. = kurtosis. ** *p* < 0.01; Gender: 0 = male, 1 = female; Age: measured in years; Tenure: measured in years; Contact: percentage of time spent in direct contact with patients.

**Table 2:** Results of expected CFA and alternative models in Study 1.

STUDY 1—PANDEMIC TIMES								
Model	χ^2^	df	*p*	RMSEA	RMSEA [90% CI]	SRMR	CFI	TLI
3-factor model ^d^	27.96	19	0.00	0.05	[0.00, 0.09]	0.05	0.99	0.98
3-factor model ^c^	48.59	24	0.00	0.08	[0.05, 0.11]	0.07	0.97	0.95
2-factor model ^b^	240.12	26	0.00	0.22	[0.20, 0.25]	0.13	0.71	0.60
1-factor model ^a^	342.97	27	0.00	0.26	[0.24, 0.29]	0.26	0.58	0.44

Note. Df = degree of freedom; RMSEA = Root Mean Square Error of Approximation; SRMR = Standardized Root Mean Square Residuals; CFI = Comparative Fit Index; TLI = Tucker–Lewis Index. ^a^ all indicators load on a single factor. ^b^ patient aggression, and cynicism load on the first factor, self-efficacy loads on the second factor. ^c^ patient aggression, cynicism, and self-efficacy load on different factors. ^d^ previous model with the addition with the inclusion of a common method latent variable on which makes all the items loaded.

**Table 3:** Results of moderated mediation model while controlling for covariates in Study 1.

AIC	BIC		
4398.83	4540.29		
Effects	*B*	*S.E.*	*95% CI*
CVA → CYN	0.64 ***	0.08	[0.47, 0.77]
CYN → SAT	−1.07 ***	0.08	[−1.22, −0.92]
CVA → SAT	−0.52 ***	0.11	[−0.81, −0.29]
WSE → CYN	−0.32 ***	0.08	[−0.48, −0.11]
CVA*WSE → CYN	−0.00	0.11	[−0.22, 0.29]
Gender → CVA	−0.06	0.08	[−0.22, 0.15]
Age → CVA	0.08	0.11	[−0.14, 0.36]
Tenure → CVA	−0.03	0.11	[−0.25, 0.25]
Contact → CVA	0.15	0.08	[−0.01, 0.36]
Gender → CYN	−0.13	0.07	[−0.26, 0.04]
Age → CYN	−0.01	0.09	[−0.18, 0.22]
Tenure → CYN	0.01	0.09	[−0.16, 0.25]
Contact → CYN	−0.05	0.07	[−0.18, 0.12]
Gender → SAT	−0.06	0.05	[−0.17, 0.08]
Age → SAT	−0.15 *	0.07	[−0.29, −0.00]
Tenure → SAT	0.03	0.07	[−0.11, 0.22]
Contact → SAT	−0.06	0.06	[−0.17, 0.08]
CVA*Low WSE → CYN → SAT	−1.27 ***	0.27	[−1.81, −0.73]
CVA*Mod WSE → CYN → SAT	−1.27 ***	0.27	[−1.80, −0.73]
CVA*High WSE → CYN → SAT	−1.26 **	0.41	[−2.05, −0.46]

Note. CVA = patient verbal aggression; CYN = cynicism; SAT = satisfaction; WSE = work-related self-efficacy; Gender: 0 = male, 1 = female; Age: measured in years; Tenure: measured in years; Contact: percentage of time spent in direct contact with patients; * *p* < 0.05, ** *p* < 0.01, and *** *p* < 0.001.

**Table 4:** Descriptive statistics and correlations among variables in Study 2.

STUDY 2—POST-PANDEMIC TIMES													
	M	SD	Sk.	Kur.	(1)	(2)	(3)	(4)	(5)	(6)	(7)	(8)	(9)
1. Aggr.	1.75	0.52	0.92	1.09	**0.88**								
2. Cynis.	1.82	1.01	1.11	0.23	0.25 **	**0.80**							
3. Sat.	3.48	1.67	-	-	0.23 **	0.55 **	-						
4. SE	2.58	1.68	0.26	−0.99	0.32 **	0.58 **	0.41 **	**0.92**					
5. Sex	-	-	-	-	0.02	−0.01	−0.04	−0.03	-				
6. Age	48.22	10.96	-	-	−0.11 **	−0.05	0.04	−0.15 **	−0.09	-			
7. Ten.	21.36	12.01	-	-	0.10	0.18 **	0.23 *	0.20 **	−0.16 **	−0.04	-		
8. Contact	66.71	32.13	-	-	−0.02	−0.02	0.10	−0.08	−0.09	−0.04	0.01		
9. Wload	2.80	0.86	0.17	−0.51	0.26 **	0.30 *	−0.36 **	−0.30 **	0.05	0.03	0.05	−0.03	**0.78**

Note. Boldfaced numbers on the diagonal represent Cronbach’s alpha; Aggr. = user verbal aggression, Cynis. = mental distance, Sat. = job satisfaction, SE. = RESE-NE, Wload = workload, M = mean, SD = standard deviation, Sk. = skewness, Kur. = kurtosis. * *p* < 0.05 and ** *p* < 0.01; Gender: 0 = male, 1 = female; Age: measured in years; Tenure: measured in years; Contact: percentage of time spent in direct contact with patients.

**Table 5:** Results of expected CFA and alternative models in Study 2.

POST-PANDEMIC TIMES								
Model	χ^2^	df	*p*	RMSEA	RMSEA [90% CI]	SRMR	CFI	TLI
4-factor model ^d^	1588.87	206	0.00	0.07	[0.07, 0.08]	0.19	0.90	0.88
4-factor model ^g^	1499.49	224	0.00	0.07	[0.06, 0.07]	0.05	0.91	0.90
3-factor model ^f^	3542.63	227	0.00	0.11	[0.11, 0.11]	0.11	0.77	0.74
2-factor model ^e^	5531.38	229	0.00	0.14	[0.14, 0.14]	0.13	0.63	0.59
1-factor model ^a^	8457.29	230	0.00	0.17	[0.17, 0.18]	0.16	0.42	0.37

Note. df = degree of freedom; RMSEA = Root Mean Square Error of Approximation; SRMR = Standardized Root Mean Square Residuals; CFI = Comparative Fit Index; TLI = Tucker–Lewis Index. ^a^ all indicators load on a single factor. ^d^ previous model with the addition with the inclusion of a common method latent variable which makes all the items loaded. ^e^ user aggression, cynicism, and workload load on the first factor, and self-efficacy loads on the second factor. ^f^ user aggression and cynicism load on the first factor, self-efficacy loads on the second factor, and workload loads on the third factor. ^g^ user aggression, cynicism, workload, and self-efficacy load on different factors.

**Table 6:** Results of moderated mediation model while controlling for covariates in Study 2.

AIC	BIC		
48,036.41	48,391.13		
Effects	*B*	*S.E.*	*95% CI*
CVA → CYN	0.24 ***	0.03	[0.17, 0.30]
CYN → SAT	−0.56 ***	0.02	[−0.61, −0.51]
CVA → SAT	−0.03	0.03	[−0.08, 0.03]
RESE → CYN	−0.033 ***	0.04	[−0.41, −0.26]
CVA*RESE → CYN	−0.07 *	0.03	[−0.13, −0.01]
Gender → CVA	0.05	0.03	[−0.01, 0.11]
Age → CVA	−0.03	0.06	[−0.14, 0.08]
Tenure → CVA	−0.11 *	0.06	[−0.22, −0.01]
Contact → CVA	0.15 ***	0.03	[0.09, 0.30]
Gender → CYN	−0.09 **	0.03	[−0.14, −0.03]
Age → CYN	−0.08	0.05	[−0.19, 0.02]
Tenure → CYN	0.019 ***	0.05	[0.08, 0.30]
Contact → CYN	−0.03	0.03	[−0.08, 0.03]
Gender → SAT	−0.07 **	0.02	[−0.12, −0.02]
Age → SAT	0.00	0.05	[−0.09, 0.09]
Tenure → SAT	−0.03	0.05	[−0.12, 0.07]
Contact → SAT	−0.00	0.03	[−0.05, 0.05]
CVA*Low RESE → CYN → SAT	−0.48 ***	0.07	[−0.62, −0.34]
CVA*Mod RESE → CYN → SAT	−0.37 ***	0.05	[−0.48, −0.26]
CVA*High RESE → CYN → SAT	−0.26 **	0.08	[−0.41, −0.10]

Note. CVA = user verbal aggression; CYN = mental distance; SAT = satisfaction; RESE= regulatory emotional self-efficacy; Gender: 0 = male, 1 = female; Age: measured in years; Tenure: measured in years; Contact: percentage of time spent in direct contact with patients; * *p* < 0.05, ** *p* < 0.01, and *** *p* < 0.001.

**Table 7:** Path coefficients and conditional effects for the moderated mediation model.

Paths	Effects		
	*B*	*S.E.*	*95% CI*
CVA → CYN	0.21 ***	0.04	[0.13, 0.28]
CYN → SAT	−1.06 ***	0.06	[−1.19, −0.94]
CVA → SAT	0.03	0.06	[−0.09, 0.15]
RESE → CYN	−0.30 ***	0.04	[−0.37, −0.22]
CVA*RESE → CYN	−0.10 **	0.04	[−0.17, −0.02]
WLD → SAT	−0.39 ***	0.05	[−0.50, −0.29]
CYN*WLD → SAT	−0.12 *	0.06	[−0.23, −0.01]
Contact → CVA	0.01 ***	0.00	[0.00, 0.01]
Tenure → CVA	−0.01 **	0.00	[−0.01, −0.00]
Gender → CYN	−0.21 **	0.07	[−0.36, −0.07]
Tenure → CYN	0.01 ***	0.00	[0.00, 0.01]
Gender → SAT	−0.29 *	0.11	[−0.51, −0.06]
CVA*Low RESE → CYN*Low WLD → SAT	−0.28 ***	0.05	[−0.39, −0.18]
CVA*Low RESE→ CYN*Mod WLD → SAT	−0.32 ***	0.05	[−0.43, −0.22]
CVA*Low RESE→ CYN*High WLD → SAT	−0.36 ***	0.06	[−0.47, −0.24]
CVA*Mod RESE→ CYN*Low WLD → SAT	−0.19 ***	0.04	[−0.27, −0.11]
CVA*Mod RESE→ CYN*Mod WLD → SAT	−0.22 ***	0.04	[−0.30, −0.14]
CVA*Mod RESE→ CYN*High WLD → SAT	−0.24 ***	0.05	[−0.34, −0.15]
CVA*High RESE → CYN*Low WLD → SAT	−0.10	0.05	[−0.21, 0.00]
CVA*High RESE → CYN*Mod WLD → SAT	−0.12	0.06	[−0.24, 0.00]
CVA*High RESE → CYN*High WLD → SAT	−0.13	0.07	[−0.27, 0.00]

Note. CVA = user verbal aggression; CYN = mental distance; SAT = satisfaction; RESE = regulatory emotional self-efficacy; WLD = workload; Gender: 0 = male, 1 = female; Age: measured in years; Tenure: measured in years; Contact: percentage of time spent in direct contact with patients; * *p* < 0.05, ** *p* < 0.01, and *** *p* < 0.001.

## Data Availability

Data that supports the findings of this study are available upon reasonable request from the first authors.
